# Anti-CD24 bio Modified PEGylated Gold Nanoparticles as Targeted Computed Tomography Contrast Agent

**DOI:** 10.15171/apb.2018.068

**Published:** 2018-11-29

**Authors:** Mona Fazel Ghaziyani, Mohammad Pourhassan Moghaddam, Daryoush Shahbazi-Gahrouei, Mostafa Ghavami, Ali Mohammadi, Mehran Mesgari Abbasi, Behzad Baradaran

**Affiliations:** ^1^Department of Medical Physics, School of Medicine, Isfahan University of Medical Sciences, Isfahan, Iran.; ^2^Immunology Research Center, Tabriz University of Medical Sciences, Tabriz, Iran.; ^3^Department of Medical Biotechnology, Faculty of Advanced Medical Sciences, Tabriz University of Medical Sciences, Tabriz, Iran.; ^4^Department of Radiology, Paramedical School, Tabriz University of Medical Sciences, Tabriz, Iran.; ^5^Drug Applied Research Center, Tabriz University of Medical Sciences, Tabriz, Iran.

**Keywords:** Gold nanoparticle, CD24 antibody, Computed tomography, Cancer imaging

## Abstract

***Purpose:*** Molecular imaging is one of the import methods for recognition of cancer at the early stage in order to enhance the capacity of remedy. This study was aimed to introduce a new contrast agent that was targeted with CD24 so as to improve the CT scan detection of cancer cells with higher CD24 expression.

***Methods:*** The surface modifications of gold nanoparticles (Au-NPs) were done with long PEG (HS-PEG-CH3O) and short PEG (HS-PEG-COOH) chains to enhance their stability and capacity for immobilization of different antibodies. MTT assay was carried out to assess the biocompatibility of the NPs. The obtained contrast agent was implemented in the targeted CT imaging based on in vitro and in vivo studies of breast cancer.

***Results:*** The results revealed that the attached CD24 to the cell surface of PEGylated Au-NPs could enhance significantly the cells CT number (40.45 HU in 4T1, while it was 16.61 HU in CT26) It was shown that the attenuation coefﬁcient of the molecularly targeted cells was more than 2 times excessive than the control groups. Further, the tumor region in model of xenograft tumor has higher density compare to the omnipaque groups, 60 min after injection (45 Hu vs.81 Hu). These results showed that the nanoparticles stayed in tumor region for longer time.

***Conclusion:*** It is predicted that the synthesized nanoparticle can be used as computed tomography contrast agent. Also, it can be used to identify the tumor cells with higher expression of CD24 at the early stages more efficiently compare to the other routine methods.

## Introduction

Cancer imaging is mainly classified into two groups: (i) anatomical imaging and (ii) molecular imaging.^1^ Computed Tomography (CT) is an anatomical imaging method that can be applied in clinical section for detection of different steps in cancer progress such as tumor site or propagation.^2^ CT as an effective diagnostic imaging system has different properties, including:; inexpensive, deeply tissue permeation, density resolution and great spatial. CT contrast agents were used basically to improve the contrast among tissues with the similar or lower attenuation by raising the signal/noise rate without increment of the radiation dose to the patient.^3-5^ The most common contrast agents that are clinically used are iodinated molecules and barium sulfate suspension. However, they have different drawbacks such as renal toxicity, deficiency in the targeting process and insufficient circulation time, which limit their application. These problems are related to the misleading outputs in diagnostic progress. Therefore, designing the appropriate contrast operator with limited defects and higher sensitivity/selectivity could be helpful for cancer detection at the early stages with higher accuracy.^1,5-7^

Gold nanoparticles (Au-NPs) have been considered for decades as a CT contrast agent. The scientific and clinical interest to use Au-NPs as CT contrast agents have steadily grown due to having various favorable characterizations. It is reported that gold is able to display higher CT attenuation coefficient compare to the iodine barium sulfate suspension, relatively.^8-11^ Moreover, since Au-NPs have higher molecular weight compare to the other contrast agents, which is associated with the increment of image window availability, they reveal a longer vascular retention time. Also, the surface functionalization of Au-NPs could improve their stability and the capacity of molecular imaging.^12-16^ It was previously shown that Au-NPs can be easily conjugated with different targets, especially antibodies due to having tunable surface. , Polyethylene glycol (PEG) can be used to enhance the stability of Au-NPs in the physiological conditions and also prevent aggregation of the NPs.^17,18^

CD24 (cluster of differentiation 24) is known as a surface marker that is highly expressed in the various cancer cells such as renal carcinoma, hepatocellular, nasopharyngeal, bladder, ovarian cancer, breast cancer and lung cancer.^19-23^ It was previously reported that CD24 is strongly associated with the capacity of metastasis in the solid tumors via P-selection interactions. It is suggested that the detection of CD24 as a crucial biomarker can help to improve the cancer treatment procedure.^24-26^

In the present study, we designed a targeted CD24-PEGylated Au-NPs to improve the cancer detection in both *in vitro* and *in vivo* investigations. This study introduced a new contrast agent with different favorable properties for application in CT molecular imaging.

## Materials and Methods

PEG monomethyl ether with a carboxyl end group (HS-PEG-COOH; MW = 3500), polyethylene glycol (HS-PEG-CH3O; MW = 6000), 1-Ethyl-3-(3 – dimethylaminopropyl) carbodiimide (EDC), N-hydroxysuccinimide (NHS) and 3-(4, 5-dimethyl-thiazol-2-yl)-2, 5-diphenyl tetrazolium bromide (MTT) purchased from sigma Aldrich (Shanghai, China). Tetrachloroauric (III) acid trihydrate (HAuCl4) and all other chemicals materials were obtained from Merck (Germany). 4T1 and CT26 cells were supplied from Pasteur Institute (Tehran, Iran). RPMI-1640 medium, fetal bovine serum (FBS), penicillin, and streptomycin were collected from GipcoBRL Company. The used water in all experiments was prepared by using a Milli-Q plus 185 water purification systems (Millipore, Bedford, MA).

### Chemical synthesis, Bio conjugation of Au NP

Frist of all, citrate reduction technique was used to chemical synthesis.^27^ In order to cover the synthesized Au-NPs, a mixture of long PEG (HS-PEG-OMe; MW = 6000) and short PEG (HS-PEG-COOH; MW = 3500) with a molar ratio of 1 to 3 was incubated with a 50 ml of Au-NPs in water solution for 72 h. The provided solution was centrifuged at 5000 rpm for 5 min and then washed three times to remove the unbound PEG chains. Then, 3 μL of carbodiimide hydrochhloride (EDC) (0.4 M) and (N-hydroxysuccinimide) NHS (0.1 M) were added to the final volume of PEGylated nanoparticle depositions (100 μL) and incubated for 10 min so as to active the carboxylate groups of PEG terminal. The final samples were centrifuged at 3000 rpm for 3 min and repeated it for 5 times, and then redisposed with PBS buffer (80 μL) to eliminate EDC/NHS molecules. Afterward, 20 μL of CD24 (0.5 mg/mL) was added to the mixture and incubation for 2 hours in room temperature to provide CD24-PEGlatedAu NPs. After centrifugation at 4 °C and 2000 rpm for 5 min .The attached antibodies to AuNPs were then blocked with 1% BSA in PBST buffer and kept at 4 °C for further uses. In this study the nanoparticles concentration investigated with a formula and in the following we design a standard curve for investigated the concentration of nanoparticle with their OD.^28^

### Characterization technique 

The successful synthesis and bioconjugation of AuNPs were monitored from 400 nm to 700 nm by UV-Vis spectroscopy. TEM (Transmission electron microscopy) imaging (2010F JEOL analytical electron microscope, Japan) was implemented to assess the size and morphology of the nanoparticles. 5 mL of the samples from the aqueous solution was dropped onto a carbon-coated copper grid to be dried for further measurements. The particle size distribution and mean particle diameter were studied with DLS (Malvern Zetasizer Nano ZSP).

### Dot-blot assay

1µl of 4T1 and CT26 cell lysis with equal total protein were directly spotted onto the nitrocellulose membrane. The spots were dried and the membrane was rinsed with PBS buffer. Then, they were blocked with 3% BSA and kept at 37°C for 1 h. Subsequently, the membranes were incubated with CD24-PEGylated AuNPs and IgG-PEGylated AuNPs as the control groups for 2 h at room temperature.

### Cell culture

Mouse cell lines, including 4T1 and CT26 were obtained from Pasteur institute, Tehran, Iran. The provided cells were incessantly cultured in RPMI 1640 medium that contained FBS (fetal bovine serum) (10%) and penicillin/streptomycin (1%). Finally, the cultured cells were sustained in a humidified atmosphere at 37 ºC with 5% CO_2_.

### Evaluation of the biocompatibility using MTT assay

To identify the biocompatibility of synthesized CD24-PEGlatedAu NPs, a tetrazolium dye MTT assay was carried out. As a summery, both of the cell cultures were trypsinized at the early step of log phase, and then cultured in 96-well plates (10^4^ cells/well/200 µl). Following that, they were incubated (37 °C and 5% CO_2_) for 24h, and then replaced with a fresh medium that contained various amounts of CD24-PEG-Au NPs (0, 0.005, 0.01, 0.02, 0.05 and 0.1 mM) for 4 and 24 h. One group that treated with only CD24 as control group. Afterward, by addition 50 μl MTT (2 mg/mL) to the supplied wells, individually, they were incubated in a humidified atmosphere for 4h based on the manufacturer’s instructions. DMSO (dimethyl sulfoxide) and Sorenson buffer (25 µL) were used to solubilize the formazan crystals. ELISA plate reader (Bio Teck, Germany) was used to monitor the absorbance of it at 570 nm. All tests were repeated at least three times. In this study, the absorbance peak was used to determine the concentrations, and also the cell viability percentage according to the following formula:

Percentage cell viability (%) = (mean absorbance of test/mean absorbance of control) × 100.

### Flow cytometry

Flow cytometry analyses of the two cancer cell lines (4T1 and CT26) were performed to evaluate the expression of CD24 as a surface marker. 4T1 and CT26 cell lines were incubated in the absence of serum during 24 h, before ﬂow cytometry assessments. The aimed cells were trypsinized and washed twice with ﬂow-activated cell sorter (FACS)-buffer (PBS supplemented with 0.5% bovine serum albumin, 0.01% NaN3, and 0.35 mM EDTA). Finally, they were resuspended in FACS buffer. For each analysis, at least 10^6^ cells were stained by using mouse anti- rat CD24 antibody. As the secondary antibody of goat, the coupled anti-rat with FITC (fluorescein isothiocyanate) (2; Dako) was used to detect the rate of CD24 expression. To be ensure about the nonspeciﬁc staining, all analyses were also conducted by the stained cells through the appropriate isotope control antibodies. Then, the cell lines were analyzed through a FACS Caliber ﬂow cytometer (Becton Dickinson, Mountain View, CA). Data are presented as the difference between the average ﬂuorescence intensities of the specified stain and non-specified stain cells.

### CT number measurement 

In order to provide a successive dilutions (10 samples) of PEGylated Au-NPs and iohexol (Omnipaque®, 300 mg iodine per mL, GE Healthcare, Milwaukee, WI, USA), various amounts of [Au] or [I] (0.005 - 1 mM) were prepared in the micro tubes (1.5 mL). Then, the provided solutions were placed into the scanning holder. Further, to normalize the absorption of X-ray, distilled water was used. The sample tubes were scanned by using a 16 row multi detector CT system (Bright Speed VCT, GE Medical Systems, Milwaukee, WI, USA) according to the these settings: tube current: 150 mA; tube voltage: 120 kV; slice space: 0 slice thickness: 0.625 mm. Then, GE imaging workstation (Advantage Workstation 4.3, GE Medical Systems, and Milwaukee, WI, USA) was used to measure CT number. Furthermore, to measure the CT value, a circle (5 mm) as region of interest was set during the imaging process in five different slices. The results were represented as mean ± standard deviation.

### In vitro CT imaging of cancer cells

4T1 cells were cultured (1.5 × 10^6^ cells/well) in a 6-well plate one day. Then, the medium was replaced and incubated with a fresh medium that contained CD24-PEGylated Au-NPs (0.1 mM) for 3 h at 37 ºC and 5% CO_2_. Furthermore, the incubated CT26 cells with CD24-PEGlated Au-NPs, and incubated 4T1 cell with IgG-PEGylated-Au NPs were considered as control samples at the same condition. After washing the cells with PBS for 5 min, they were trypsinzed and then centrifuged. Following that, the samples were resuspended in PBS (200 µL) into Eppendorf tubes (0.5 mL). Also, the same concentrations of water in Eppendorf tube (0.5 mL) was used as a control sample to normalize the absorption. The obtained tubes were placed into a designed scanning holder and then scanned based on the mentioned CT imaging system. CT number measurement was done according previously study protocol.^29^

### Targeted Computed Tomography imaging of 4T1 tumor in vivo

All animal cares were done based on the protocol that is approved by the institutional committee and also in accordance with the policy of the National Ministry. In the first stage, female BALB/C mice (4-6-week-old) (18-20 g, Pasteur Institute, Tehran, Iran) were injected subcutaneously in the abdominal mammary gland (1 × 10^6^ cells/mouse). After approximately 2 weeks of the injection, the volumes of tumor nodules were reached to 0.7-1.2 cm^3^. The considered mice were classify to 3 groups (each group was three mice) Then, they were anesthetized by injection of Xylazine 2% (0.2 mL) and Ketamine hydrochl, intraperitoneally. Following that, they were intravenously injected with CD24 –PEGylated Au-NPs and omnipaque (100 mL, [Au] 1 mM), and placed into a scanning holder. The injected mice with CD24 –PEGylated Au-NPs and omnipaque were then scanned 15 and 60 min after injection. Next, the images were reconstructed on CT imaging workstation (Advantage Workstation 4.3, GE Medical Systems, and Milwaukee, WI, USA). Further, CT scanning was carried out before the injection as control images according to the same protocol and CT number was measured.^29^ Fundamental guidelines for the care and use of laboratory animals were followed in dealing with the animals.

### Statistical analysis

Data were presented as means ± SE from at least three independent experiments and analyzed by Student‘s t-test. Differences at p <0.05 were considered statistically significant.

## Results and Discussion

### Synthesis and Characterization of CD24 modified Au-NPs 

**Figure 1:**
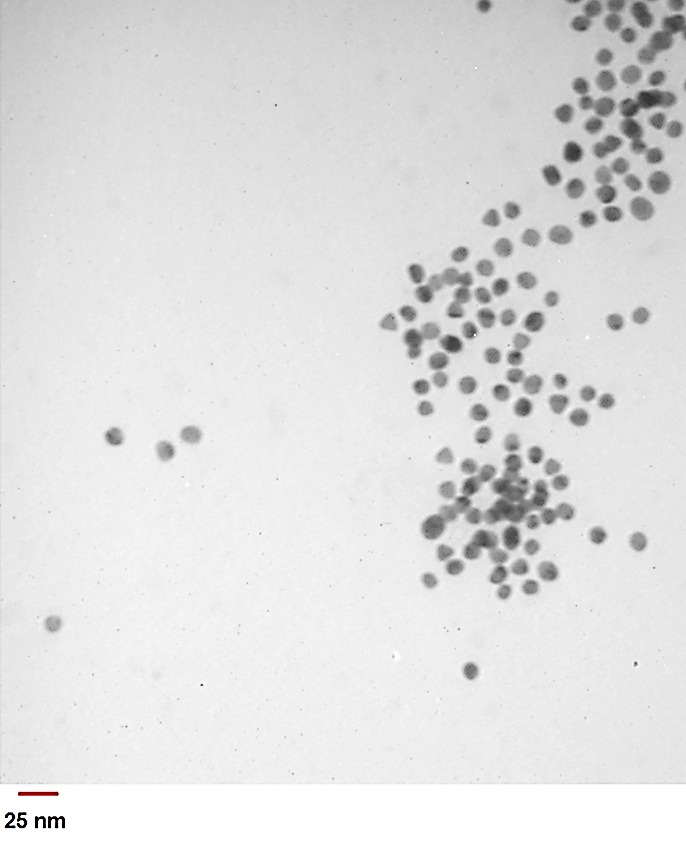
TEM (Transmission electron microscopy) image of gold nanoparticles; the gold nanoparticles were observed to be 10-15 nm in diameter and as spherical shapes on the TEM image

**Figure 2:**
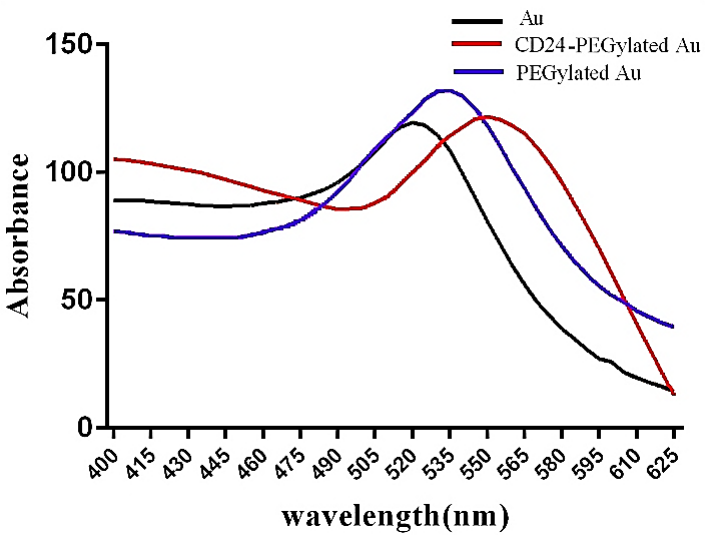
UV–visible spectra of Au NP, PEGylated AuNPs and CD24- PEGylated-Au NP. The maximum absorption shift indicates that PEGylated gold nanoparticles conjugated with antiCD24

To provide a desirable nanoparticles that contain appropriate characterizations as a contrast agent of CT, PEG/ m-PEG (long/short) chains were used to cover gold nanoparticles so as to enhance their biocompatibility and reduce their aggregation.^1,30,31^ Previously, it was reported that by addition of PEG to Au-NPs, they are able to rise the circulation time, which is essential for the imaging application.^32-34^ Further, covering of Au-NPs with PEG to could affect in the NPs size, which is related to the modulation of EPR (full name) effects. It is also associated with the enhancement of solubility in buffer due to having hydrophilic ethylene glycol repeats.^34^ The covered NPs with PEG and m-PEG can be easily conjugated with different antibodies.^19^ Also, the terminal groups of PEG are able to be activated by functional groups such as EDC/NHS that have activator of COOH groups and activate COOH of PEG chain to conjugate with NH2 of antibody (CD24). By attachment of functional groups to the NPs, the modified NPs would obtain some unique characteristics, including biocompatibility, appropriate size and maximum wave length, which is useful for *in vitro* and *in vivo* assays. The transmission electron microscopy of CD24-PEGylated AuNPs indicated that the particles are uniformly dispersed with a significant narrow size range of 15–20 nm. The average particle size was estimated to be 13 nm. Also, it was shown that Au-NPs contain a spherical shape in their structures (Figure 1). UV–vis absorption spectra of AuNPs, PEGylated AuNPs and CD24-PEGylated AuNPs are presented in Figure 2. The unmodified nanoparticles displayed characteristic of surface Plasmon absorption at 525 nm, while covered nanoparticles with PEG showed a surface Plasmon band around 535 nm. However, it was indicated that the maximum absorption of surface Plasmon band was increased and shifted to 555 nm in the conjugated PEGylated AuNPs with CD24. This shift was accrued due to cause the interaction of the nanoparticles and biomolecules.

**Figure 3:**
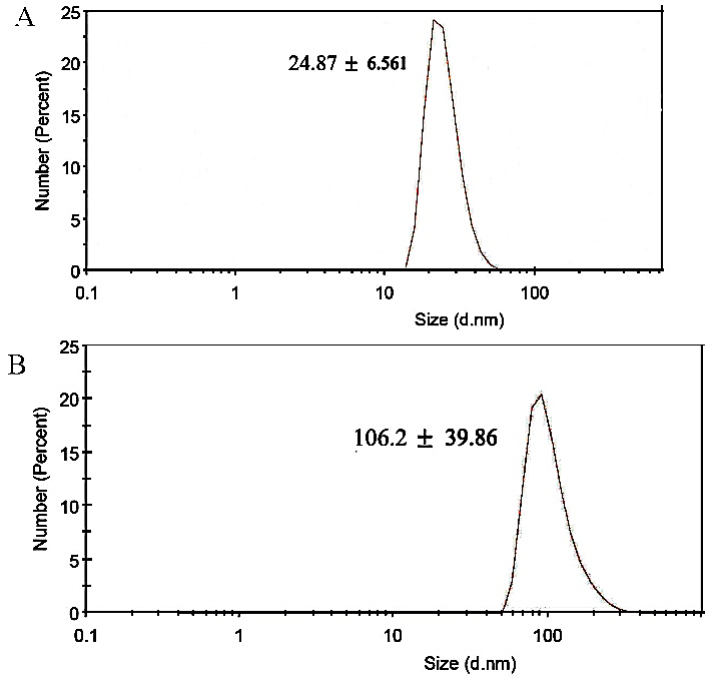
Hydrodynamic diameter of AuNPs (a) and CD24-PEGylated AuNPs (b)

The hydrodynamic diameters of AuNPs and CD24-PEGylated AuNPs were measured at pH 7.0 to further verify of the presence of antiCD24 on the gold nanoparticles. Figure 3 shows that the hydrodynamic diameter of AuNPs is smaller than CD24-PEGylated AuNPs This phenomenon might be associated with the following reasons: (i) addition of PEG and antiCD24 to the surface of AuNPs could increase the particle size (ii) the amine and carboxyl groups of antiCD24 and activated PEG groups could attract the counter ions from the solution to form electrical double layer. Therefore, the increased double layer thickness results in a larger particle diameter.

### Dot blot assay

**Figure 4:**
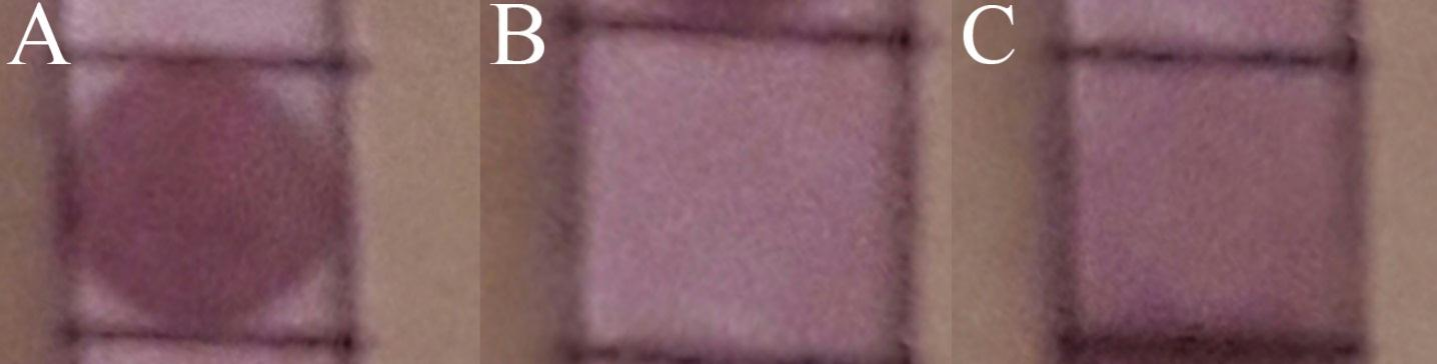
I mages of nitrocellulose membranes spotted with 4T1 cell lysis (a) and CT26 cell lysis (b) after soaking with CD24-PEGylated AuNPs, membranes spotted with 4T1 cell lysis after soaking with IgG-PEGylated AuNPs (c)

In order to use the system for detection of CD24 in the cell lysis by the prepared nanoparticle, a simple method adapted from Dot blot Elisa was applied.^35^ After spotting of 4T1 and CT26 cell lysis onto nitrocellulose membrane, the membrane was rinsed with CD24-PEGylated AuNPs. A violet spot appeared (Figure 4a) at the paper with 4T1 cell lysis while we have any spot on paper with CT26 cell lysis (Figure 4b). The obvious spot on paper indicated that CD24-PEGylated AuNPs is able to detect CD24 antibody among other proteins. Also, it proved that the synthesis of nanoparticle was well and antiCD24. attached to gold nanoparticle, correctly. Moreover, the correct connection of antiCD24 and nanoparticles were checked by using one unspecific antibody (IgG) as a targeted agent. Figure 4c shows that there was not any spot, when paper with 4T1 cell lysisi was rinsed with IgG-PEGylated AuNPs. These results showed that CD24 was connected to the nanoparticle via heavy part and its arms that attached to cell were free.

### Cytotoxicity assay 

**Figure 5:**
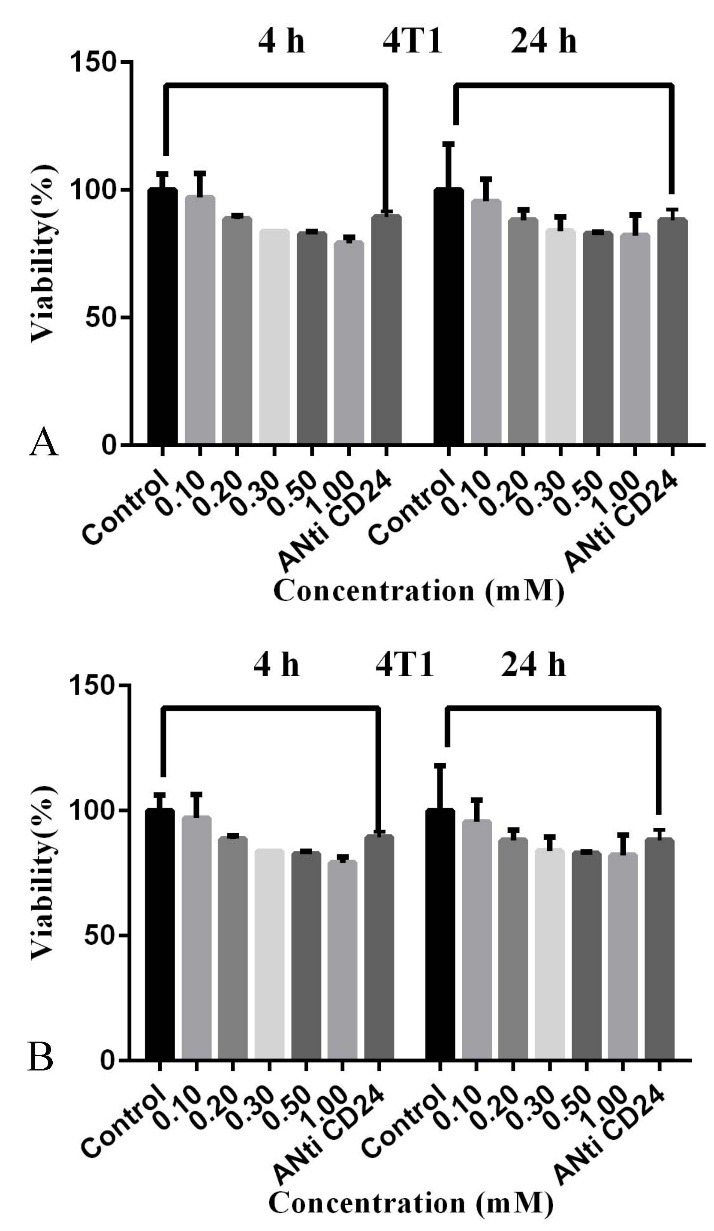
Viability of 4T1 cells (a) and CT26 cells (b) after treatment with CD24-PEGylatedAu NPs at the different Au concentrations for 4 and 24 h

In order to identify the cytotoxicity effects of CD24-PEGlated Au-NPs on the cells, MTT assays were done to analyze the viability of the studied cells (4T1 and CT26) in the presence of a fixed concentration of CD24-PEGlated-Au NPs (0.1 mM) for 4 and 24h. As revealed in Figures 5a and 5b, there was not a significant change in the various amounts of Au in comparison to the treated control samples (P > 0.05) with PBS buffer. The results were same for both cell lines. The achieved results displayed that CD24-PEGlated Au-NPs are compatible in various amounts of Au in the biological condition.

### X-ray attenuation property of NPs 

**Figure 6:**
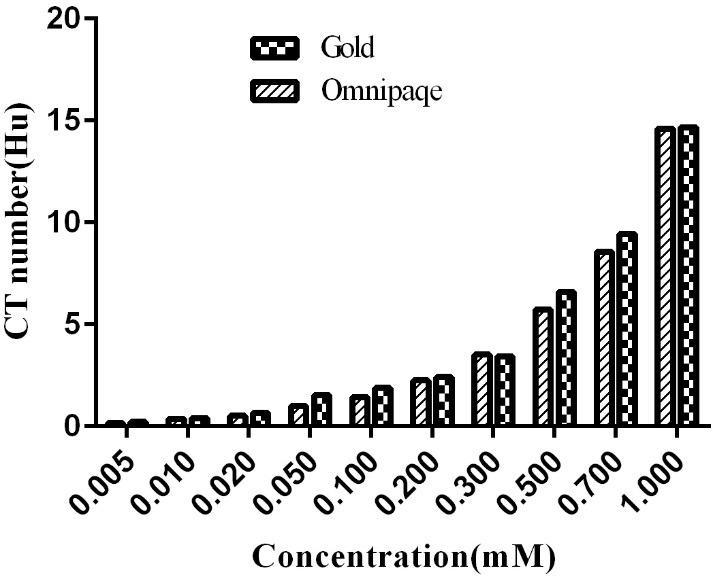
CT images (a) and X-ray attenuation (HU) intensity (b) of PEGylated Au NPs and omnipaque as a function of the mM concentration of radio dense element (Au and iodine)

It is shown that gold atoms have a greater X-ray absorption coefficient compare to other elements in the contrast agent such as iodine due to cause higher electron density and atomic number.^1^ Some reports could verify this theory and indicated differences in the attenuation coefficient in the concentrations greater than 0.01M.^30^ In this work, the X-ray attenuation coefficient of PEGylated Au-NPs was assessed and then compared with the different dilutions of Omnipaque (ten samples). Meanwhile, the absorption of water sample was used to normalize the absorption data. The amounts of Au and iodine in all samples were less than 1 mM. Figure 6 shows that by rising of the amounts of Au and I, the attenuation coefficients of X-ray in both PEGylated Au-NPs and omnipaque groups were considerably increased based according to the quantitative study. It is considerable that, the X-ray attenuation rate was steadily higher than iohexol in the presence of PEGylated Au-NPs. According to the capacity of CD24 conjugated PEGylated Au-NPs, the difference in the amounts of Au and I (lower than 1 mM) was implemented in the radiation absorption so as to apply in computing tomography.

### In vitro CT imaging of cancer cells

**Figure 7:**
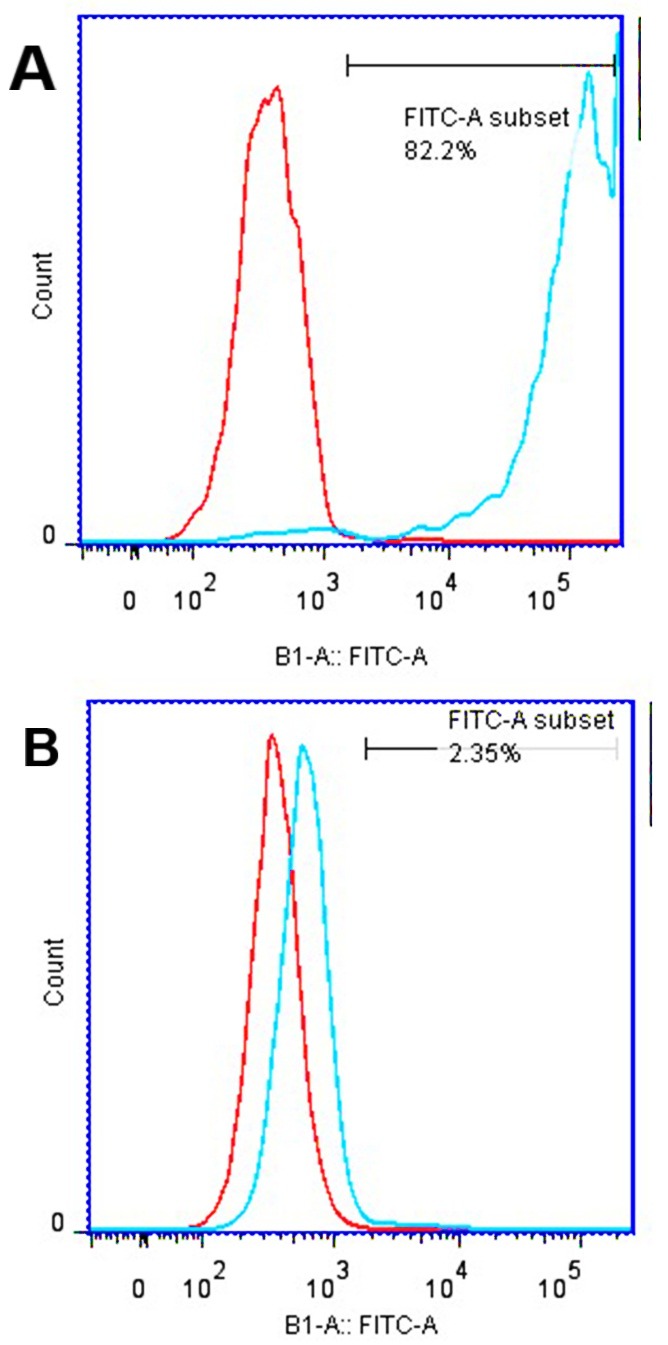
CD24 expression on 4T1 cells and (a) and CT26 cells (b)

**Figure 8:**
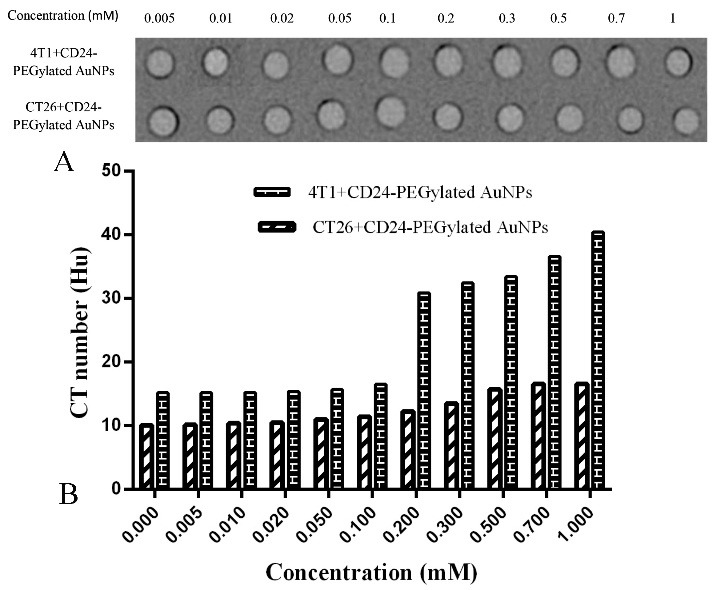
CT images (a) and x-ray attenuation intensity (b) of 4T1 and CT26 cell lines after incubation with CD24-PEGylated AuNPs with different concentration

**Figure 9:**
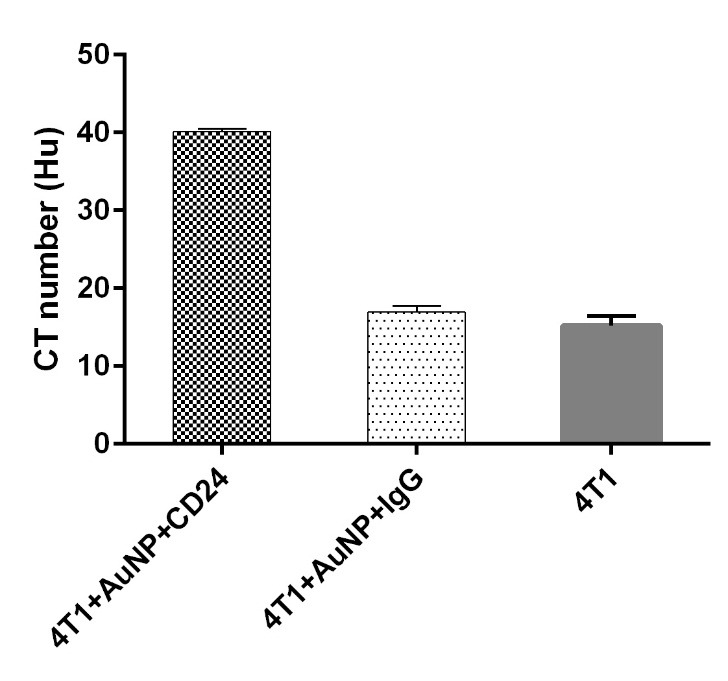
x-ray attenuation of 4T1 cell line after incubation with IgG-PEGylated AuNPs and PEGylated Au NPs

For the purpose of computed tomography image of cells with targeted contrast media, it is very important to know the amount of CD24 expression on the cells surface. Evaluation of CD24 Expression on 4T1 and CT26 cell lines was done with flow cytomety. Conjugated secondary anti body with FITC was used to detect CD24 on cells surface. As it is reported in Figure7a, the flow cytometry results of 4T1 cells showed a considerable overexpression of CD24 (approximately 80%), but CT26 cell lines express CD24, rarely (Figure 7b). CT imaging was done for both of the cell lines (4T1 and CT26 cells), which were incubated by CD24-PEGlated Au-NPs. Also for investigated correct connection of antibody to nanoparticle 4T1 cells incubated with IgG-PEGylated AuNPs to define function of antibody. Since the detection of brightness difference of 4T1 and CT26 cells is difficult in CT images (Figure 8a), the CT number of the provided images was analyzed based on quantitative analyses. As revealed in Figure 8b, the obtained attenuation values from CT imaging application was shown in Hounsfield unit (HU). From the results, the signal (HU that achieved from the targeted 4T1 cells) was dramatically higher than the control values (HU that achieved from the targeted CT26 cells). Further, the attenuation coefﬁcient of the targeted 4T1 cells in higher concentration was calculated to be 40.45 HU, while it was 16.61 HU to the targeted CT26 cells. These results indicated that CD24-PEGlatedAuNPs were attached obviously to 4T1 cells. Considering the attenuation values of the control samples (negative), the nonspeciﬁc bindings were rare in the studied samples, comparatively. It is suggested that attachment of CD24-PEGylated AuNPs to 4T1 cells is the major cause of the increment of the radiation absorption. Also difference between CT number of 4T1 cell that incubated with IgG-AuNPs and CD24-AuNPs (16.95 Hu and 40.14 Hu, respectively) proved dot blot assay result and prove correct connection of antibody to gold nanoparticle. (Figure 9)

### Targeted CT imaging of 4T1 cells in vivo

**Figure 10:**
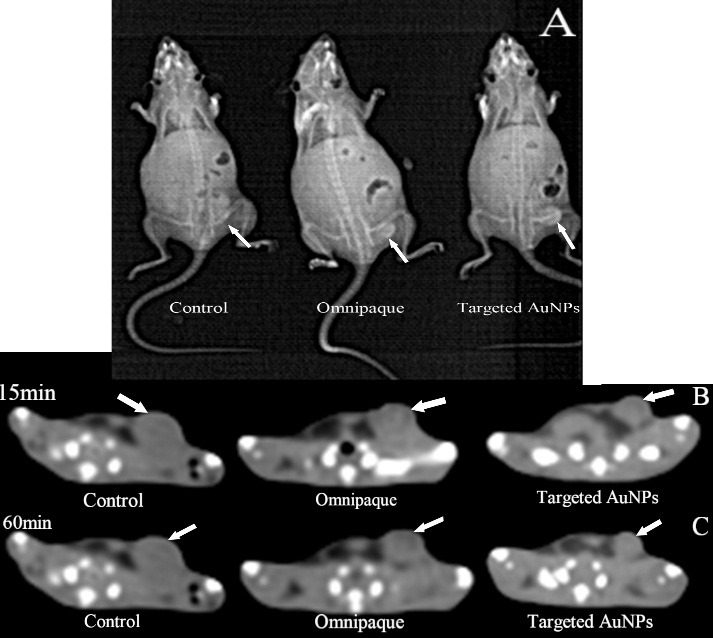
Scan gram CT of mice bearing 4T1 tumor before and after intravenous injection of the contrast agent (A) CT image of mice bearing 4T1 tumor after intravenous injection of the prepared nanoparticles at 15 and 60 min post injection (B) CT image of mice were injected with omnipaque at 15 and 60 min post injection (C).white arrow showed the tumor site

**Figure 11:**
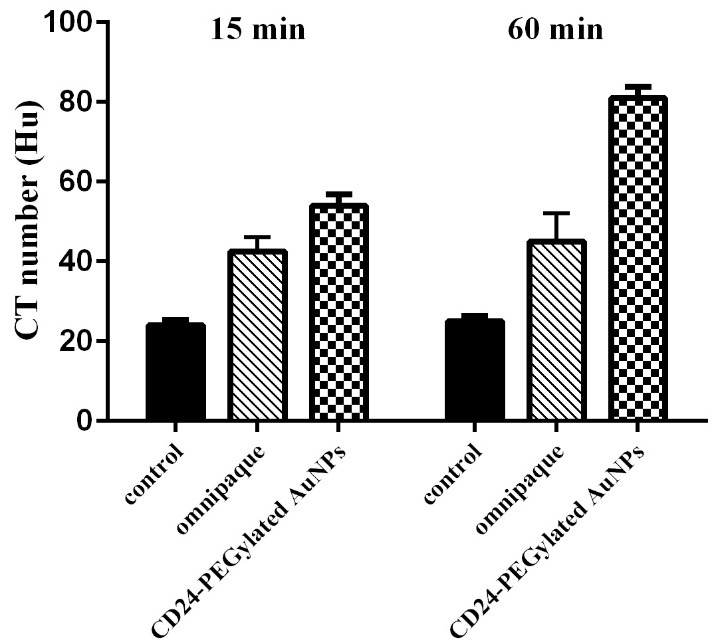
CT value in Hounsfield unit (HU) of the tumor region before injection and at 15 and 60 min post injection

The anatomical structure is a significant difference between normal vessels and tumor nonvascular vessels.‏ Since the formation of tumor vessels is rapidly though an unregulated system in comparison with the normal vessel, so a considerable rise in the gaps between endothelial cells of vascular in tumor vessels can be seen. Therefore, nanoparticles with the size smaller than 150 nm can be passed easily from the walls of tumor vessel, while it would not be occurred in the normal tissues.^7^ The modified AuNPs with the appropriate biocompatibility that are targeted by specific CD24 antibodies can be an applicable method for recognition of cancer in different stages. In this study, the medical applicability of CD24-PEGylated AuNPs was explored by CT imaging of the cell line (4T1 cells) in *in vivo* experiments. The detection of different cancers in early stages is a crucial factor for implementation of effective therapeutic application; therefore, a small xenografted model (0.7-1.2 cm^3^) was induced in BALB/C mice in order to assess the capacity of the NPs. To this purpose, CT number of the induced tumor was investigated before and after injection in 15 and 60 min, which are common minimum and maximum times in CT scan protocols, respectively. Because of the low amount of contrast agent concentration (1mM), it was not detectable in qualitative CT (Figure 10a,Figure 10b). For this reason, quantitative CT was considered; this method showed that the mean CT number of each slice increases after injection, also for identify the rate of the enhancement we consider a circle (5 mm) in tumor reign in five different slices to determine tumor reign mean CT number (Figure 11). Based on the collected CT images of the induced tumor, a significant enhancement in the rate of CT value was obviously shown in the injected CD24-PEGylated AuNPs animals, intravenously at different time in comparison with the control group (P < 0.05；Figure 11). Further, a significant difference was observed between the tumor sites that were injected, individually with CD24-PEGylateg gold nanoparticles and omnipaque at 60 min (45 Hu vs.81 Hu). These result represented that the more concentrations of the Nano contrast in the tumor site compared to the omnipaque-injected samples. This findings maybe is associated with the attachment of CD24-PEGylated AuNPs to 4T1 cells. The obtained results obviously showed that CD24-PEGylated AuNPs is a reliable Nano contrast to detect breast tumors through CT imaging application.

## Conclusion

Biomarkers are known as potential and inexpensive applications for cancer studies, including screening, risk assessment, identification of prognosis, differential diagnosis, recognition of the treatment process, and also monitoring cancer stages. Therefore, biomarkers have important roles in identification cancer stages. Development of different quantitative tools for assessment of biomarkers in the cancer cells not only can help to facilitate detection of cancer stages to treat effectively, but also can help to recognize the best therapeutic agents. In this study, CD24 antibody as well-known biomarker was thoroughly conjugated to the modified gold nanoparticle (CD24-PEGylated Au-NPs ) for recognition of the cancer, and also implemented as a targeted Nano contrast to detect breast cancer cells ( 4T1 cells) in both *in vitro* and *in vivo* experiments. The results of this research indicated that the introduced Nano contrast could be helpful for detection of the cancer cells, which highly express CD24 antibody based on clinical application of CT scans.

## Acknowledgments

This work is a part of PhD thesis which financially (Grant No: 395550) supported by Isfahan University of Medical Sciences, Isfahan, Iran.

## Ethical Issues

Ethical approval was obtained from the ethics committee of the Isfahan University of Medical Sciences (ethics code: 395550)

## Conflict of Interest

All authors declare no conflicts of interest.
